# Comparison of Diagnostic Accuracy between Octopus 900 and Goldmann Kinetic Visual Fields

**DOI:** 10.1155/2014/214829

**Published:** 2014-01-23

**Authors:** Fiona J. Rowe, Alison Rowlands

**Affiliations:** ^1^Department of Health Services Research, Whelan Building, University of Liverpool, Brownlow Hill, Liverpool L69 3GB, UK; ^2^Department of Ophthalmology, Warrington and Halton Hospitals NHS Foundation Trust, Lovely Lane, Warrington WA5 1QG, UK

## Abstract

*Purpose*. To determine diagnostic accuracy of kinetic visual field assessment by Octopus 900 perimetry compared with Goldmann perimetry. *Methods*. Prospective cross section evaluation of 40 control subjects with full visual fields and 50 patients with known visual field loss. Comparison of test duration and area measurement of isopters for Octopus 3, 5, and 10°/sec stimulus speeds. Comparison of test duration and type of visual field classification for Octopus versus Goldmann perimetry. Results were independently graded for presence/absence of field defect and for type and location of defect. Statistical evaluation comprised of ANOVA and paired t test for evaluation of parametric data with Bonferroni adjustment. Bland Altman and Kappa tests were used for measurement of agreement between data. *Results*. Octopus 5°/sec perimetry had comparable test duration to Goldmann perimetry. Octopus perimetry reliably detected type and location of visual field loss with visual fields matched to Goldmann results in 88.8% of results (*K* = 0.775). *Conclusions*. Kinetic perimetry requires individual tailoring to ensure accuracy. Octopus perimetry was reproducible for presence/absence of visual field defect. Our screening protocol when using Octopus perimetry is 5°/sec for determining boundaries of peripheral isopters and 3°/sec for blind spot mapping with further evaluation of area of field loss for defect depth and size.

## 1. Introduction

Goldmann kinetic perimetry has been an essential perimetry assessment in many areas of ophthalmology practice including the assessment of young children, patients with poor vision or severely restricted visual fields, and patients with brain injury involving the posterior hemispheres of the brain [1–5]. The Goldmann perimeter is primarily a kinetic perimetry and uses a mobile target of fixed luminosity with which positions of equal sensitivity are plotted perpendicularly to the limits of the expected visual fields or isopters through the free movement of the target in all directions [6]. Goldmann perimetry is a three variable visual field examination with consideration of background illumination, stimulus luminosity, and stimulus size. The results differ depending on photopic, mesopic, or scotopic background luminosity. It allows for good control of fixation with direct viewing of the patient's eye by the examiner using the built-in telescope [7]. Due to the continuous, customized operator/patient interaction, the diagnostic accuracy of Goldmann kinetic perimetry remains of high value in many perimetry examinations.

In 2007, the production of the Goldmann perimeter ceased. Its replacement is the Octopus 900 perimeter (Haag Streit International, Koeniz, Switzerland). The Octopus 900 provides 90 degree full field projection perimetry with a range of 47 decibels and capable of performing both kinetic and static perimetry programmes. The vectors chosen for kinetic perimetry can be preselected and run as an automatic test or performed “live” choosing vectors for assessment dependent on patient responses during the test. When performed “live,” the perimeter is being utilised in the same way as Goldmann kinetic perimetry.

Comparison of semiautomated kinetic perimetry (Octopus 101 perimeter) has been undertaken against Goldmann kinetic perimetry. Semiautomated kinetic perimetry produced similar results for detection and localisation of area of visual field deficit and was deemed reliable and reproducible [7, 8].

Although the Octopus 900 perimeter provides age/matched comparisons from its data bank from which comparisons can be made of new visual field results, there is little data available in the literature on the comparison of kinetic visual field results obtained by Goldmann versus Octopus 900 perimetry. Our research questions asked what variances might occur in visual field area, in a standardised photopic setting, when using different stimulus test speeds on Octopus perimetry, and what is the diagnostic accuracy of Octopus perimetry when used in a semiautomated mode in comparison to the “gold standard” Goldmann kinetic perimetry which is a purely manual mode of operation. This was determined in a group of healthy controls with full visual fields and in a further group of patients with visual field impairment.

## 2. Methods and Materials

A prospective cross section survey was undertaken with local ethical approval and in accordance with the Tenets of the Declaration of Helsinki.

### 2.1. Subjects

Subjects with full visual fields were recruited from the hospital department staff (there was a high female preponderance in department employees). Inclusion criteria included adults of 18 years or older, having cognitive and motor ability sufficient to perform the tests and willingness to undergo standard kinetic tests on both perimeters on the same day. Exclusion criteria included a visual acuity of worse than 0.1 logMAR, history of ocular disease such as glaucoma, and the patient being unable to complete kinetic perimetry for both eyes using both perimeters within the same testing session.

Patients with visual field loss referred for assessment of visual field regardless of their ocular diagnosis were recruited. Of note, patients referred for kinetic visual field assessment were those usually with diagnoses of neurological and posterior visual pathway damage such as idiopathic intracranial hypertension, functional/malingering suspects, pituitary adenoma, stroke, and brain trauma injuries. Participants were identified randomly; that is, notes were taken consecutively from the list waiting for visual field assessment without prior knowledge of patient ability and cognition. A selection bias existed in that the patients recruited to this study had been booked in to an out-patient visual field clinic for kinetic perimetry. Thus, there was an assumption that these patients had sufficient ability and cognition to undertake standard perimetry.

Inclusion criteria were adult patients aged 18 years or older attending for visual field assessment, sufficient motor ability to sit at the perimeter unaided, able to press the response button, sufficient cognitive ability to understand and follow instructions for performing the test, and willingness to undergo standard kinetic tests on both perimeters on the same day. The exclusion criteria were patients with visual acuity less than 1.0 logMAR, who were unable to sit at the perimeter, unable to follow instructions for performing the test, or too ill to complete the full assessment. All patients underwent perimetry following full explanation of the purpose of the test and procedure.

### 2.2. Visual Field Assessment

Full (normal) visual fields by kinetic assessment were defined as visual field results with isopters for I4e and I2e falling within age-matched ranges and no focal defects within the isopter area (apart from the blind spot in the temporal field). Visual field loss was defined as isopter boundaries constricted within the age-matched ranges which could be global constriction or a defect type. Defect types were classified according to a modified list based on those reported by Pineles et al. [8] and the Ocular Hypertension Treatment Study (OHTS) [9] and outlined in Table 1. We added a category of functional visual field loss where the visual field defect followed a tubular or spiral pattern on testing. Functional visual field loss was considered present in patients in whom the remainder of their ocular examination was normal and no explanation could be found for their visual field loss, that is, nonorganic visual field loss.

The study protocol consisted of visual field assessment with both Goldmann perimetry and Octopus perimetry on the same day. Perimetry was undertaken by the same examiner for standardisation. The order of testing was randomised as to which of the two assessment types was used first and also for which eye was tested first in order to take fatigue effect in to consideration.

We defined an isopter as a curve of equal retinal sensitivity of the visual field and a vector as the direction of movement of the stimulus. The vector typically moved from the periphery to the central visual field area (centripetal movement).

**Figure 1:**
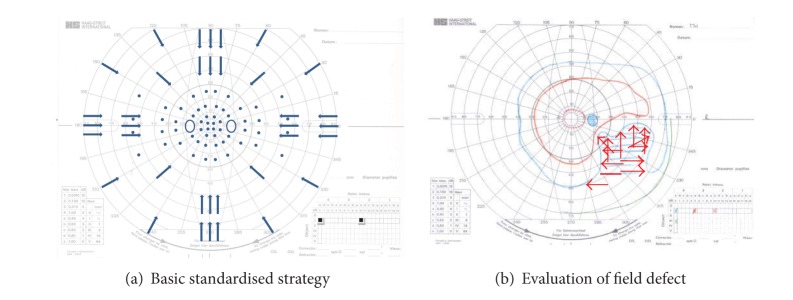
(a) Arrows indicate the trajectory of the kinetic target. Small circles indicate position of static stimuli. (b) Goldmann result showing partial right inferior quadrant loss of the right eye. Red arrows indicate additional kinetic trajectories used to define the boundary of the field loss.

**Figure 2:**
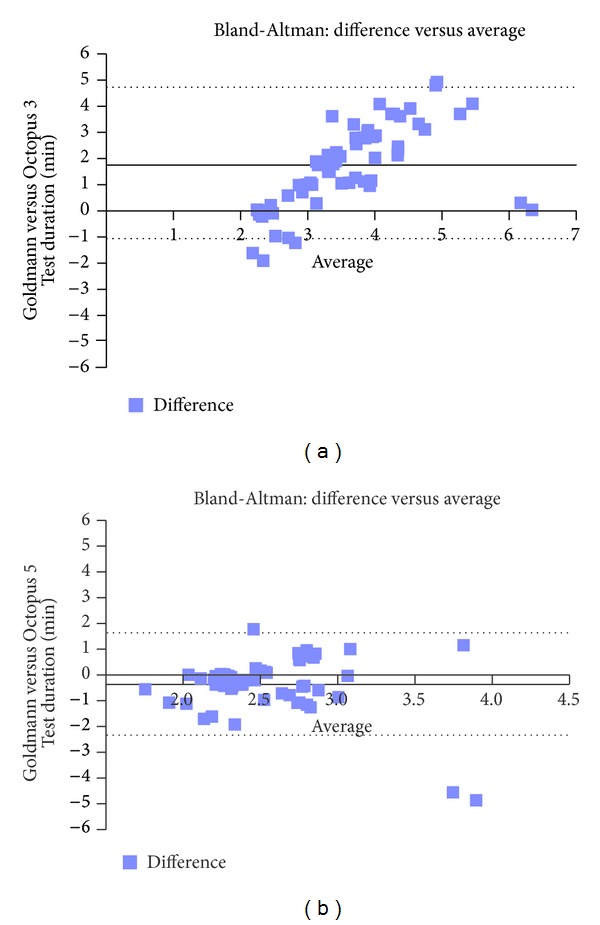
Test duration—Bland-Altman plots. (a) Example of proportional effect for comparisons to 3°/second stimulus speeds, the dotted lines represent ±1.96 SD of −1.21 to 4.73. The solid line represents the mean bias of 1.76 minutes. There is a proportional effect in which the difference in test duration between the two tests increases as the average test duration of the two tests increases. Thus, the longer the average test, the more difference occurs. (b) Example of close comparisons to 5°/second stimulus speeds, the dotted lines represent ±1.96 SD of −2.36 to 1.63. The solid line represents the mean bias of −0.36 minutes. Most differences lie close to the mean difference and within the limits of agreement.

For the purposes of basic testing standardisation and to avoid potential examiner bias, a screening protocol was used (Figure 1). The same testing strategy was utilised for both Goldmann and Octopus perimetry so that a direct comparison could be undertaken for both results. Two stimuli of the same size (0.25 mm^2^) were used but of different intensity (I4e, 1000 apostilbs and I2e, 100 apostilbs). The peripheral visual field boundary and blind spot were assessed using a size I4e target. Central visual field boundary was assessed using a size I2e target. A minimum of twelve vectors were assessed for the peripheral visual field and eight for the central visual field inclusive of vectors on and offset from the vertical and horizontal meridian moving centripetally, similar to previously reported testing strategies [10, 11]. In addition, 56 static points (14 per quadrant) were assessed within the central 30 degrees of the visual field using the I4e target (Figure 1). Where a visual field defect was found, this was further evaluated using additional vectors with direction of target movement perpendicular to the boundary of the field defect (Figure 2). Following assessment, the response points along each vector, including any additional vectors required to plot visual field deficits, were joined to form the isopter for I4e and I2e targets, respectively.

Movement of the target on the Octopus perimeter was set at 3, 5, or 10°/sec. Movement of the target on the Goldmann perimeter was approximately 5°/sec for central and peripheral isopters and 3°/sec for the blindspot. This was calculated manually by timing the distance in degrees (*d*°) measured within a 5 second time period (*x*°/sec = *d*/5). Area of visual field was calculated automatically on Octopus perimetry for each target (isopter) and the result provided as degrees^2^. Duration of assessment was assessed automatically on Octopus perimetry and manually using a stopwatch for Goldmann perimetry.

Visual field results were deemed unreliable if patient fixation was considered poor by the examiner (by observation through the Goldmann reticulated telescope or on the Octopus eye monitor) or if the blindspot could not be mapped as described previously [8].

### 2.3. Comparison of Results

Visual field results in both groups were assessed for presence or absence of visual field defects and for the former, were further assessed for type and location of visual field defect as per Table 1. One author assessed the results of Octopus perimetry (FR) and the second author assessed the results of Goldmann perimetry (AR). Each reviewer was masked to patient identifiers and to the classification by the other reviewer. Visual fields were graded as normal (full) or abnormal (field defect evident). For those graded as abnormal, results were further categorised as to type of defect (e.g., hemianopia, quadrantanopia, altitudinal, and scotoma [8, 9]). A positive match was deemed present if the category of defect was the same for corresponding Octopus and Goldmann results. The results fell into two groups: group 1 was a match with identical or similar results and group 2 was a nonmatch with dissimilar results.

### 2.4. Statistical Analysis

A direct comparison of results was made for Goldmann and Octopus perimetry results using the statistical package SPSS version 19 (IBM SPSS Statistics, USA). To evaluate normality of distribution of results from right and left eyes, a Kolmogorov-Smirnov test was used. Comparison of areas of visual field obtained for Octopus perimetry with stimulus test speeds of 3, 5, and 10°/sec was undertaken using ANOVA with Bonferroni adjustment for multiple comparisons. Analysis was undertaken for comparison of Octopus at 5°/sec versus Goldmann perimetry using unpaired *t* test. Bland-Altman plots were used to compare the differences between two independent measurements versus the average of both. Comparisons of independent grading of Octopus and Goldmann visual field results was made by Chi-square (*χ* ^2^) test and Cohen's kappa statistic (varying from 0—no agreement to 1—perfect agreement).

## 3. Results

In total, 90 subjects were recruited to this study: forty subjects with full visual fields and fifty subjects with visual field loss.

### 3.1. Comparison of Stimulus Test Speeds: Full Field

Forty subjects were recruited with full visual fields (80 eyes): 32 females and 8 males. Each subject had visual acuity of 0.1 logMAR or better in both eyes and no known ocular history apart from spectacle correction for refractive error. The mean age at assessment was 35.5 years (SD 11). When the test durations and isopter area were plotted for right and left eyes, there were no significant differences in distribution of results between the two eyes and data were therefore combined for analysis.

#### 3.1.1. Test Duration

Mean test durations and mean differences are shown in Table 2. Test duration was slowest for Octopus 3°/sec perimetry (mean 4.46 minutes, SD 1.42) but similar for Goldmann perimetry, Octopus 5°/sec perimetry, and Octopus 10°/sec perimetry (means ranging from 1.76 to 2.33 minutes). All comparisons of test duration to Octopus 3°/sec were significant (*P* = 0.0001 ANOVA). There were no significant differences when comparing Octopus 5°/sec to either Octopus 10°/sec stimulus speed or Goldmann perimetry. Bland-Altman plots showed that the greatest differences in mean difference involved comparisons to the Octopus 3°/sec stimulus speed in which a proportional effect was seen showing increasing differences between tests as the average increased (Figure 2). In comparison, the least differences were seen across increasing averages for comparisons to Octopus 5°/sec stimulus speed in which most differences were clustered near the mean bias and within low limits of agreement.

#### 3.1.2. Area of Octopus Visual Field

**Figure 3:**
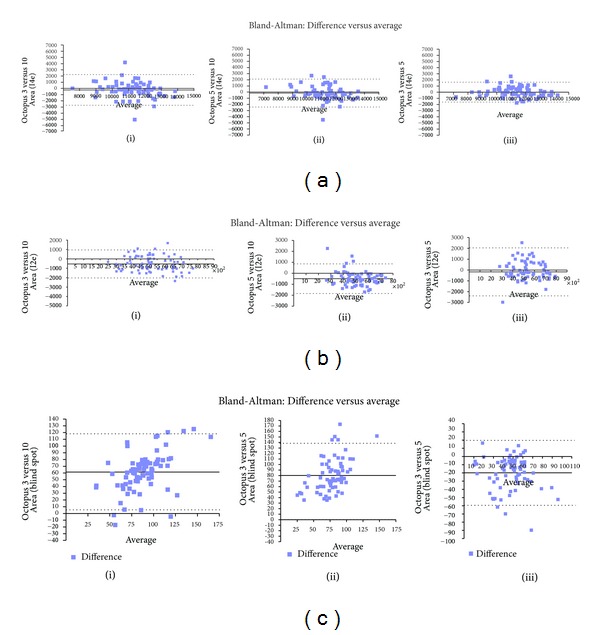
(a) Bland-Altman analysis: I4e peripheral area. (i) The dotted lines represent ±1.96SD with limits of agreement of −2731 to 2239. The solid line represents the mean bias of −246.17 degrees^2^. (ii) The dotted lines represent ±1.96SD with limits of agreement of −2433 to 2110. The solid line represents the mean bias of −161.54 degrees^2^. (iii) The dotted lines represent ±1.96SD with limits of agreement of −1601 to 1631. The solid line represents the mean bias of −15.24 degrees^2^. There is good correlation with distribution of differences across increasing average close to mean bias and within limits of agreement. (b) Bland-Altman analysis: I2e stimulus area. (i) The dotted lines represent ±1.96SD with limits of agreement of −2029 to 943. The solid line represents the mean bias of −542.81 degrees^2^. (ii) The dotted lines represent ±1.96SD with limits of agreement of −1825 to 852. The solid line represents the mean bias of −486.45 degrees^2^. The comparison of isopter area using different speeds is significant (*P* = 0.001). There is no correlation between areas obtained using different stimulus speeds with variability noted across all comparisons. (iii) The dotted lines represent ±1.96SD with limits of agreement of −1694 to 1596. The solid line represents the mean bias of −48.95 degrees^2^. (c) Bland-Altman analysis: I4e blind spot stimulus area. (i) The dotted lines represent ±1.96SD with limits of agreement of 5 to 117. The solid line represents the mean bias of 61.46 degrees^2^. The comparison of isopter area using different speeds is significant (*P* = 0.001). There is no correlation between areas obtained using different stimulus speeds with variability noted across all comparisons. (ii) The dotted lines represent ±1.96SD with limits of agreement of 22 to 138. The solid line represents the mean bias of 80.25 degrees^2^. The comparison of isopter area using different speeds is significant (*P* = 0.001). There is no correlation between areas obtained using different stimulus speeds with variability noted across all comparisons. (iii) The dotted lines represent ±1.96SD with limits of agreement of −59 to 20. The solid line represents the mean bias of −19.87 degrees^2^. The comparison of isopter area using different speeds is significant (*P* = 0.001). There is no correlation between areas obtained using different stimulus speeds with variability noted across all comparisons.

Mean isopter areas and mean differences are shown in Table 3. There were no significant differences when comparing Octopus perimetry areas at different stimulus speeds for the I4e stimulus (ANOVA with Bonferroni correction) with mean areas ranging from 11121 to 11439 degrees^2^. Bland-Altman plots showed no correlation between stimulus speeds with distribution of differences across increasing averages of area (Figure 3(a)).

For the I2e stimulus, mean areas ranged from 4856 to 5399 degrees^2^ for results obtained by different stimulus speeds. A significant difference was seen when comparing Octopus 3°/sec to 10°/sec for area, *P* = 0.05 (ANOVA with Bonferroni correction). There were no significant differences when comparing Octopus perimetry areas for 5°/sec versus 3°/sec or 10°/sec stimulus speeds. Bland-Altman plots showed no correlation between stimulus speeds with distribution of differences across increasing averages of area but with larger limits of agreement than with I4e (Figure 3(b)).

The blind spot area was determined with a I4e stimulus with mean areas ranging from 37 to 119 degrees^2^ for results obtained by different stimulus speeds. A significant difference was seen when comparing Octopus 3°/sec to 10°/sec and 5°/sec 10°/sec for area, *P* = 0.001 (ANOVA with Bonferroni correction). Bland-Altman plots showed no correlation between stimulus speeds with distribution of differences across increasing averages of area (Figure 3(c)).

### 3.2. Comparison of Octopus to Goldmann Perimetry: Visual Field Loss

Fifty subjects were recruited with evidence of visual field loss on perimetry assessment (95 eyes): 27 female and 23 males. Twenty-four patients were new to perimetry assessment and the remainder were attending for follow-up appointments. Diagnoses included cerebro-vascular accident, arteriovenous malformation, idiopathic intracranial hypertension, optic chiasm compression, and retinal lesion (Table 4). The mean age at assessment was 53 years (SD 15). Comparisons for this part of the study were made between Goldmann perimetry and Octopus 5°/sec perimetry for visual field area using I4e and I2e targets. For blind spot assessment, a 3°/sec stimulus speed was chosen for comparison to Goldmann perimetry. These stimulus speeds were chosen following analysis of data from Octopus perimetry of subjects with normal visual field results.

#### 3.2.1. Test Duration

Mean test duration was 4.26 minutes (SD 1.11) for Goldmann perimetry and 4.49 minutes (SD 1.33) for Octopus perimetry with a mean difference for Goldmann versus Octopus 5°/sec of 0.14 minute. The difference between the mean test durations was not statistically significant (*P* < 0.5, unpaired *t* test).

#### 3.2.2. Detection of Visual Field Deficit

**Figure 4:**
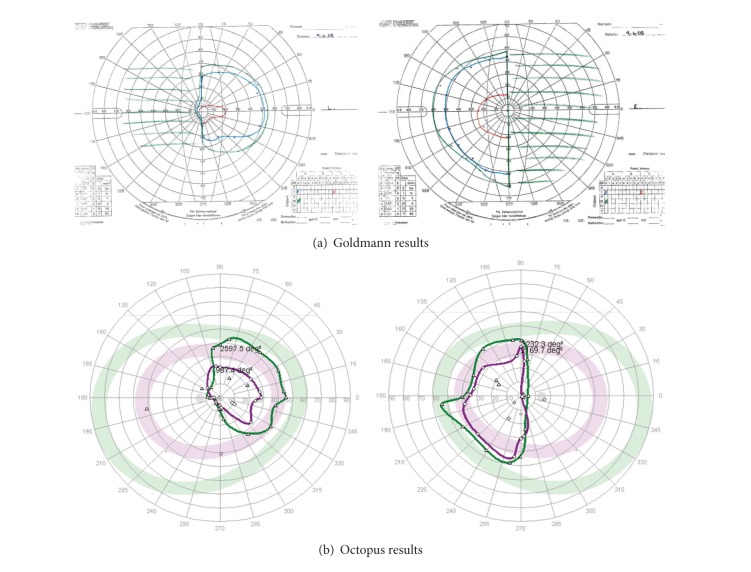
Identical/similar matched results. Patient with bitemporal hemianopia showing demarcation along vertical meridian and more extensive visual field loss in left eye compared to the right eye. Similar defect for both eyes detected on both Goldmann kinetic perimetry and Octopus semiautomated perimetry.

**Figure 5:**
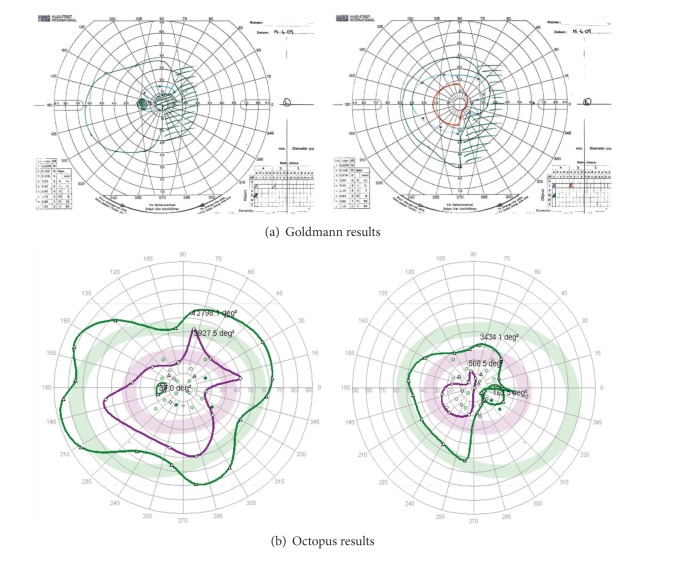
Nonidentical/dissimilar unmatched results (left eye only). Patient with right homonymous hemianopia showing a matching visual field result for the right eye on both Goldmann kinetic perimetry and Octopus semiautomated perimetry. The Goldmann result for the left eye shows the right-sided hemianopia. However, the Octopus result for the left eye does not show the defect. Although the patient maintained good central fixation, the Octopus result was felt to be due to false positive responses.

Goldmann results were graded by one observer (AR) and Octopus results graded by a second observer (FR) for location of field defect (e.g., defect in right or left side of field) and type of field defect (as per Table 1). In all patients, the location of visual field defect was identical for both perimeters (*K* = 1). The types of visual field defect classified included hemianopia (unilateral or bilateral), quadrantanopia, constricted field, full field, and superior or inferior defect (Table 4). The same type of visual field defect (group 1: identical results, e.g., Figure 4) was recorded for Octopus and Goldmann perimetry in 84 eyes which was significant (*P* = 0.0001  *χ* ^2^ test and *K* = 0.9). In the remaining eleven eyes, discrepancy of results (group 2: nonidentical results, e.g., Figure 5) between Octopus and Goldmann included (a) a mismatch in visual field defect with partial homonymous hemianopia versus constricted field (*n* = 1) and partial homonymous hemianopia versus homonymous hemianopia (*n* = 2), (b) Octopus result graded as full field versus Goldmann result showing a superior defect (*n* = 3), and (c) Goldmann result graded as full field versus Octopus results showing partial peripheral hemianopia (1) or superior defect (*n* = 1) or constricted field (*n* = 2) or inferior defect (*n* = 1). Octopus perimetry had a 96% sensitivity and 55% specificity in comparison to Goldmann perimetry (Table 5).

## 4. Discussion

In a group of subjects with full visual fields, we compared test duration of three different stimulus speeds on Octopus perimetry in comparison to Goldmann perimetry followed by comparison of area of field between the three different stimulus speeds on Octopus perimetry. No significant difference was found for test duration for the 5 and 10°/sec Octopus speeds compared to Goldmann. A significant difference was found for the 3°/sec Octopus speed versus Goldmann. Johnson et al. [12] evaluated test duration using SQUID automated perimetry and reported that the average duration of kinetic perimetry reduced significantly as the stimulus speed increased from 1 to 4°/sec. However, minimum reductions in test duration were seen at stimulus speeds greater than 4°/sec. This was similar to our findings using semiautomated Octopus perimetry. In our group of patients with visual field loss, we found no significant difference in test duration for Octopus or Goldmann kinetic perimetry. Octopus semiautomated perimetry was undertaken in a mean of 4.49 minutes using a combination of plots for two isopters (I4e and I2e) and suprathreshold static assessment of the central 30° visual field with the I4e target. Pineles and colleagues [8] used a combined strategy of the static TOP threshold central strategy overlaid on two peripheral isopters and reported a test duration ranging from 6 to 12 minutes. This was considerably longer than our test durations but most likely reflects the use of a threshold static strategy of 59 test locations versus our suprathreshold static strategy of 56 test locations.

In full visual fields using Octopus perimetry, we found that the area of visual field obtained with the 5°/sec speed did not differ significantly to the 3 and 10°/sec speeds whereas there was a significant difference between the 3 and 10°/sec speeds and particularly for the blind spot and central I2e isopter areas. A reduction in detection and thus decreased area when testing with stimulus speeds greater than 4°/sec has been reported as due to reaction times: the faster the stimulus speed, the longer the latency period from detection of the target to pressing the response button [9]. Johnson et al. [12] recommended a stimulus velocity of 4°/sec for both central and peripheral visual fields using SQUID automated perimetry. On the basis of our results, the recommendation for clinical assessment of visual fields using Octopus semiautomated perimetry includes 5°/sec speed for peripheral and central isopters but with 3°/sec speed for mapping the blindspot area using the I4e target.

Although the recommended speed when moving the Goldmann target is 2-3 degrees per second, we agree with others that there is an inherent bias effect where the target speed is faster than thought by examiners [8]. Our results showed a significant difference in test duration for Octopus 3°/sec stimulus speed when compared to Goldmann perimetry but no significant difference for Octopus 5°/sec stimulus speed compared to Goldmann. This was expected as we had manually calculated the stimulus speed using Goldmann perimetry and found this to be approximately 5°/sec.

Ramirez and colleagues [7] discussed the “technician factor” whereby bias could be introduced by alteration of speed of stimulus based on anticipation of patient response plus creation of best-fit contours for isopters. There is no bias effect using the Octopus when preset stimulus speeds are set and this may provide a more standardised method. Nevertheless, our subjects reported that the 10°/sec stimulus speed was quite fast and this may incur a degree of overestimation of rotation measurement because of delayed reactions from the point of detection of the moving target to the action of pressing the response button [12]. Subject speed of response is a factor that has been shown to result in significant inter and intraindividual variation and can be corrected for with resultant reduction in random variance [13]. Thus, we chose to compromise stimulus speed by using the 5°/sec setting. The exception to this was the use of the 3°/sec speed for plotting the blind spot area as this speed was shown in the control group to have consistent results in comparison to Goldmann perimetry and a slow stimulus speed is appropriate for determination of such a small scotomatous area. Furthermore, the 3°/sec stimulus speed was utilised when evaluating the intersection of area of visual field loss to further define defect size and depth [14].

Area of visual field has been compared for Octopus versus Goldmann perimetry in previous studies. Ramirez et al. [7] quantitatively assessed their visual field results by scanning and importing each result using computer software to ensure size equivalency between the Goldmann and Octopus results. The size corrected images were then directly compared using Scion software to quantify the isopter area. The authors also undertook qualitative assessment with grading of the results by two independent investigators. Pineles et al. [8] compared combined semiautomated and static central perimetry versus Goldmann kinetic perimetry using qualitative analysis. Their visual field results were graded by independent investigators, classified into pattern configurations (adapted from the OHTS group [9]) and matched as identical or nonidentical results.

We did not have access to software to conduct similar quantitative evaluation to that undertaken by Ramirez et al. However, our Goldmann results, when viewed qualitatively, were consistently smaller in isopter area to the Octopus results, an observation that has been reported previously [7, 15] with one study reporting that Octopus results were on average 15% larger than Goldmann kinetic perimetry and particularly for the smaller I2e target [7]. Thus we could not evaluate our results accurately without alteration for size equivalency. We chose instead to evaluate the results by qualitative analysis of classification of visual field type as undertaken by previous studies [7–9].

We compared results for Octopus versus Goldmann perimetry for subjects with visual field loss using Kappa analysis of agreement between independent observers.

Our results were classified as normal or abnormal and subsequently as defect type such as hemianopia, quadrantanopia, or scotoma. This was similar to a previous study in which the authors used a modified list of pattern configurations from the OHTS group including altitudinal, scotoma, hemianopia, and arcuate defect [8, 9]. The location of visual field defect was matched in every result. The type of visual field defect was matched in 84 of 95 eyes (88.5%) which is similar to previous studies reporting identical or similar results from automated and Goldmann perimetry in 77 to 80% [7, 8, 12, 14].

A further observation when evaluating our results is that we noted that the I2e stimulus showed the visual field defect more clearly on Octopus perimetry than with the I4e stimulus. This is most likely related to the reduced intensity of the stimulus which allows the evaluation of the relative depth of the visual field defect. We believe our results confirm the suitability of utilising additional small, dim target for discrimination of more subtle deficits. We used a standardised strategy for assessment of the visual field but allowing for additional vectors to be added to further evaluate the boundaries and depth of any detected area of visual field loss. This adds to the length of time required for the visual field assessment, particularly for patients with anterior visual pathway damage, but does show the value of starting the test with a standardised “template” and amending this further as indicated.

A limitation of this study is that one perimetrist undertook all visual field assessment. Order of testing with Goldmann or Octopus perimetry was randomised and, for patients who underwent Goldmann kinetic perimetry after Octopus perimetry, it is possible that bias could occur in testing due to prior knowledge of the Octopus visual field result. However, when reviewing the results of Goldmann kinetic assessment tested first versus tested second, no differences were found for the latter results being better matched than the former. A further limitation of this study is that we did not repeat perimetry assessments in our study. This we are unable to report intratest variability for test duration, area of visual field, and classification of visual field defect.

In eleven eyes (11.5%), we found a mismatch of defect between perimeters and typically the field defect was not as extensive on one or other perimeter. In three results, the Octopus field was classed as normal when the Goldmann results showed a mild superior defect. It is possible that inherent bias led to the detection of the superior defect on Goldmann perimetry. However, in further five results, the Goldmann field was classed as normal when the Octopus results showed partial peripheral defects or constriction of the field. In these cases, the interpretation of the Octopus results may have been aided by the presence of the age-matched normal isopter locations on the printout.

Pineles et al. [8] also reported 8% of their results to show a mismatch in which the result was normal on one perimeter but showed a visual field defect on the other perimeter. They were unable to reach a consensus in matching the results in a further 14%. However, they proposed that their combined semiautomated and static perimetry option could be used in most cases as an alternative to standard testing to improve detection of visual field loss. They reported that minimum skill was required on the part of the examiner for their testing strategy but it did require that the examiner was computer literate and would understand perimetry sufficiently to recognise when vectors should be retested or new vectors added to further evaluate suspect areas of visual field. We agree with these comments on the basis of utilising a preprogrammed kinetic screening strategy and proposed that our semiautomated kinetic assessment strategy with additional central static point testing is a valid and reliable alternative to Goldmann kinetic perimetry. Furthermore, the Octopus perimeter provides computerised storage of all visual field results which can be viewed from remote computer terminals if networked with an easy reprint option where patient case notes are unavailable.

## 5. Conclusions

Duration of visual field assessment was similar for Goldmann perimetry and Octopus perimetry using a 5°/sec stimulus speed to plot peripheral and central isopters and the 3°/sec stimulus speed for assessment of blind spot area and for additional assessment of intersection of visual field defect area.

Octopus perimetry detected the presence of all visual field defects with strong agreement in comparison to Goldmann perimetry for type and location of defect.

Based on the results of these visual field assessments, the visual field strategy currently utilised when screening with our Octopus perimeter is a 5°/sec stimulus speed for peripheral and central visual field isopters using I4e and I2e targets along with a 3°/sec stimulus speed using I4e target for blind spot mapping and further evaluation of field loss area. This is coupled with suprathreshold static assessment within the central visual field using the I4e target.

## Figures and Tables

**Table 1:** Classification of visual field abnormalities.

Visual field classification	Number of results (total 95 eyes)	
	Octopus perimetry	Goldmann perimetry
Normal	9	11
Altitudinal defect		
Arcuate defect		
Constriction (widespread)	17	15
Functional		
Homonymous hemianopia	37	36
Bitemporal hemianopia	4	4
Inferior defect	1	0
Nasal step		
Quadrantanopia (inferior)	19	19
Quadrantanopia (superior)	7	7
Scotoma (central)		
Scotoma (paracentral)		
Superior defect	1	3
Temporal wedge		
Vertical step		

**Table 2:** Mean test durations and mean differences.

Perimeter option	Mean test duration (minutes)	SD
Goldmann	2.73	0.71
Octopus 3°/sec	4.46	1.42
Octopus 5°/sec	2.33	0.55
Octopus 10°/sec	2.30	0.62

Perimeter comparison	Mean differences (minutes)	Significance (ANOVA: Bonferroni correction)

Goldmann-Octopus 3°/sec	1.76	*P* = 0.0001
Goldmann-Octopus 5°/sec	−0.36	*P* < 0.5 not significant
Goldmann-Octopus 10°/sec	−0.46	*P* = 0.05
Octopus 3–5°/sec	−2.04	*P* = 0.0001
Octopus 3–10°/sec	−2.33	*P* = 0.0001
Octopus 5–10°/sec	−0.09	*P* < 0.5 not significant

**Table 3:** Isopter areas for Octopus perimetry.

Perimeter option	Mean isopter area (I4e) (degrees^2^)	SD
Octopus 3°/sec	11364.02	1488.01
Octopus 5°/sec	11439.21	1383.13
Octopus 10°/sec	11121.21	1252.12

Perimeter comparison	Mean differences (degrees^2^)	Significance (ANOVA: Bonferroni correction)

Octopus 3–5°/sec	15.24	*P* ≤ 0.5not significant
Octopus 3–10°/sec	−246.17	*P* ≤ 0.5 not significant
Octopus 5–10°/sec	−161.54	*P* ≤ 0.5 not significant

Perimeter option	Mean isopter area (I2e) (degrees^2^)	SD

Octopus 3°/sec	5399.54	1344.52
Octopus 5°/sec	5359.17	1302.54
Octopus 10°/sec	4856.73	1211.33

Perimeter comparison	Mean differences (degrees^2^)	Significance (ANOVA: Bonferroni correction)

Octopus 3–5°/sec	−48.96	Not significant
Octopus 3–10°/sec	−542.81	*P* = 0.05
Octopus 5–10°/sec	−486.45	Not significant

Perimeter option	Mean isopter area (I4e blind spot) (degrees^2^)	SD

Octopus 3°/sec	57.8	19.77
Octopus 5°/sec	37.67	18.64
Octopus 10°/sec	119.26	32.51

Perimeter comparison	Mean differences (degrees^2^)	Significance (ANOVA: Bonferroni correction)

Octopus 3–5°/sec	−19.88	*P* = 0.0001
Octopus 3–10°/sec	61.46	*P* = 0.0001
Octopus 5–10°/sec	80.25	*P* = 0.0001

**Table 4:** Causes and types of visual field deficit (95 eyes).

	Homonymous hemianopia		Bitemporal hemianopia		Inferior quadrantanopia		Superior quadrantanopia		Constriction		Superior defect		Inferior defect		Full visual field	
	Oct	Gold	Oct	Gold	Oct	Gold	Oct	Gold	Oct	Gold	Oct	Gold	Oct	Gold	Oct	Gold
Brain haemorrhage	14	14														
Brain infarct	23	22			19	19	7	7	10	9		2	1		4	4
Idiopathic intracranial hypertension									4	5					2	2
Intracranial tumour			4	4												
Retinal											1	1			3	3
Multiple sclerosis									1	1						
Functional									2							2

**Table 5:** Detection of presence/absence of visual field loss.

	Octopus perimetry	
	Full visual field	Abnormal visual field
Goldmann perimetry	6	5
Full visual field	True negative	False positive
Goldmann perimetry	3	81
Abnormal visual field	False negative	True positive
